# Intraperitoneal spread in uterine sarcoma following unprotected laparoscopic transvaginal uterine morcellation: a case report and literature review

**DOI:** 10.3389/fonc.2024.1434720

**Published:** 2024-08-12

**Authors:** Jianhao Sun, Xinjuan Jiao, Zhenzhen Wu, Tingting Yao, Shumei Tuo, Yueyuan Wang, Ruirong Chen, Jing He, Jifang Qian, Shengfang Xu, Qing Liu

**Affiliations:** ^1^ Gansu Provincial Maternity and Child-care Hospital, Lanzhou, Gansu, China; ^2^ Clinical Medical College, Yangzhou University, Yangzhou, Jiangsu, China; ^3^ Qingyang Second People’s Hospital, Qingyang, Gansu, China

**Keywords:** uterine sarcomas, morcellation, laparoscopy, undifferentiated sarcomas, tumor spread

## Abstract

Clinically and through auxiliary examinations, distinguishing uterine leiomyoma from early-stage uterine sarcoma presents significant challenges. A 48-year-old patient underwent a laparoscopic hysterectomy for uterine leiomyoma, during which a large uterus was excised through the vagina and extracted. Four months post-operation, the patient developed abdominal distension, indicative of extensive pelvic-abdominal dissemination of uterine sarcoma. We hypothesize that unprotected fibroid fragmentation increases the risk of uterine sarcoma spread, thereby worsening the prognosis. Our literature review aims to thoroughly understand the risks associated with unprotected transvaginal laparoscopic tumor division.

## 1 Introduction

With advancements in laparoscopic techniques, an increasing number of patients with uterine fibroids are opting for laparoscopic surgery. In 2014, the U.S. Food and Drug Administration (FDA) issued a warning against the use of power morcellation in laparoscopic uterine fibroid surgeries. Specifically, they discouraged removing the uterus or uterine fibroids via laparoscopic power morcellation, particularly in patients with suspected or confirmed uterine malignancy (1). Uterine sarcoma, a rare malignant tumor, comprises approximately 1% of female reproductive tract malignancies and 3% to 7% of uterine malignancies. With an incidence rate of 0.36 per 100,000, it is most prevalent among women aged 45 to 55. It is highly malignant, prone to recurrence, distant metastasis, and associated with a poor prognosis (2, 3). According to the World Health Organization (WHO) classification of tumors, uterine sarcoma is derived from mesenchymal tissue and primarily includes uterine leiomyosarcoma (LMS), endometrial stromal sarcoma (ESS), and undifferentiated uterine sarcoma (UUS) (4). Previous studies reported that between 0.29% and 0.63% of patients initially diagnosed with leiomyoma and treated with laparoscopic surgery were found to have uterine sarcoma upon postoperative pathological examination (5–7). Previously, case reports of tumor spread due to morcellation primarily focused on laparoscopic myomectomy or subtotal hysterectomy (8, 9), with rare documentation of cases involving morcellation through vaginal extraction. In this report, we discuss a case of extensive pelvic and abdominal sarcoma dissemination following laparoscopic transvaginal uterine fragmentation, along with a review of related literature. The patient provided written informed consent for the publication of her information anonymously.

## 2 Case presentation

**Figure 1:**
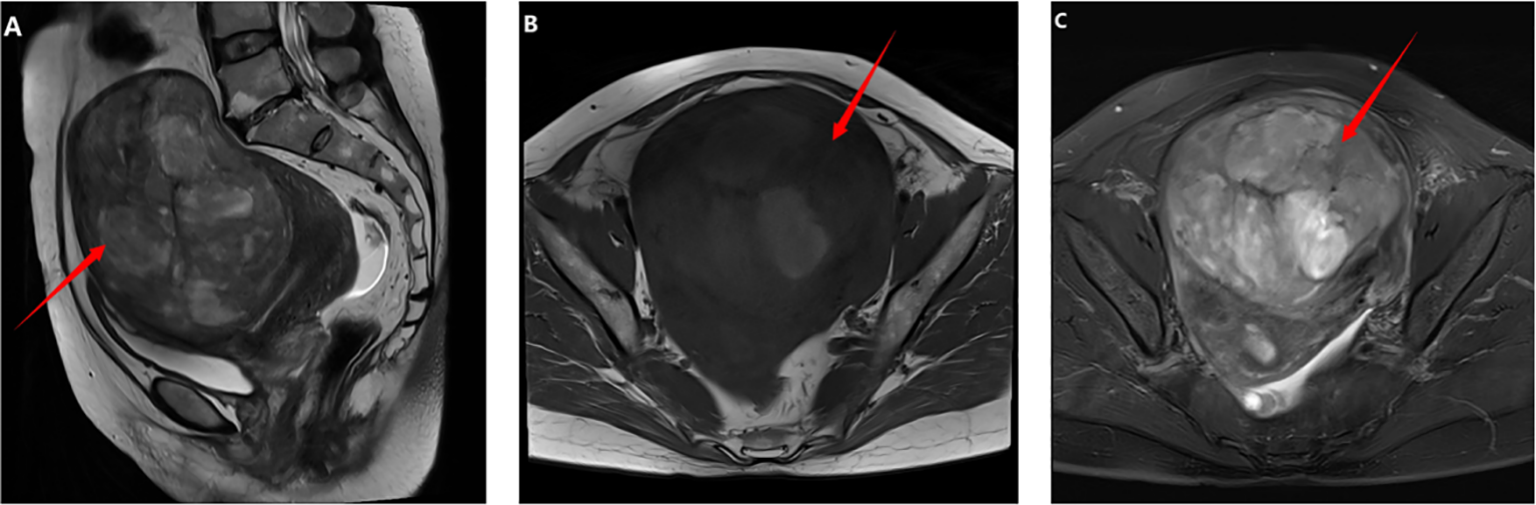
Imaging findings before the first operation: **(A–C)** Pelvic MRI revealed a tumor measuring approximately 9.9x12.0x12.6 cm in the anterior wall of the uterus. On T1-weighted images, the tumor was isointense with areas of hyperintensity. T2-weighted and fat-suppressed images showed hypointensity with areas of hyperintensity.

In December 2021, a 48-year-old woman was admitted to our center for a pelvic mass initially identified seven years earlier by transabdominal ultrasonography, which suggested a leiomyoma. Over the last three years, her menstrual cycles have lengthened, and her flow has increased. She has experienced abdominal pain and noticeable abdominal enlargement over the past five months. Following her hospitalization, a comprehensive preoperative evaluation was conducted. All serum tumor markers, including carbohydrate antigen 12-5 (CA12-5), CA19-9, alpha-fetoprotein (AFP), human epididymis protein 4 (HE4), and carcinoembryonic antigen (CEA), were within normal limits. However, her serum lactate dehydrogenase (LDH) level was elevated to 289U/L. Ultrasonography identified a heterogeneously echogenic mass in the anterior wall of the uterus measuring 12.6x12.0x8.4cm with a regular outline and clear boundaries, consistent with a uterine fibroid. It also revealed endometrial thickening to 15mm. MRI imaging displayed two quasi-circular masses within the uterine muscle wall; the larger was located in the anterior wall, measuring approximately 9.9x12.0x12.6cm, with 80% protruding into the subserosal area. The MRI suggested degeneration of the leiomyomas, possibly indicating a cellular leiomyoma (**Figure 1**). An endometrial biopsy was performed, which indicated a secretory-responsive endometrium.

**Figure 2:**
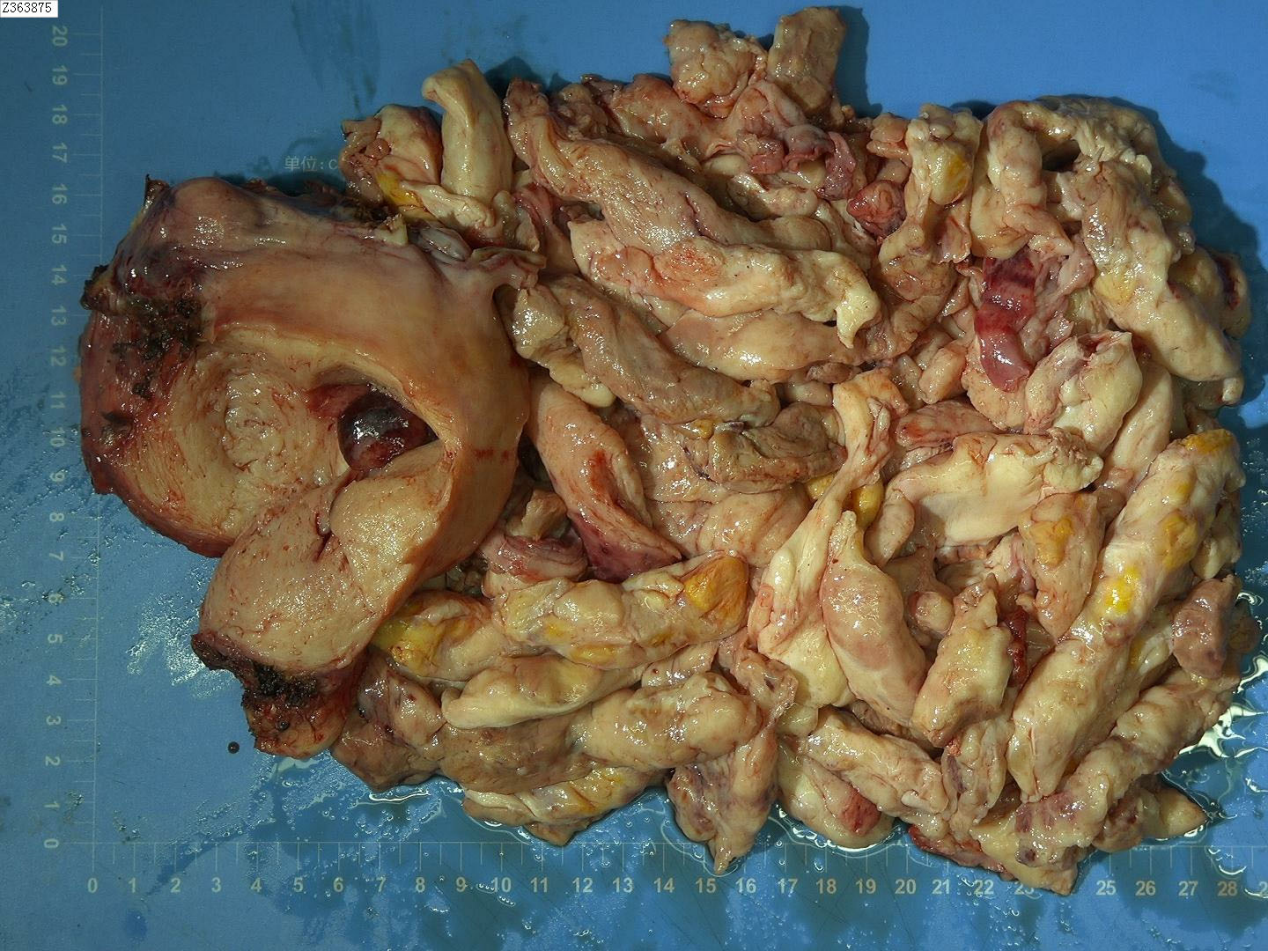
General view after the first operation. The fragmented uterus measured 15x9x4 cm. Between the muscle layers, two grayish-white nodular masses ranging from 1.5 to 3 cm in diameter were identified. These masses had tough, vortex-like textures upon sectioning. Additionally, a cluster of cylindrical tissue, grayish-white to grayish-red in color and measuring 20x20x2 cm, was discovered. This tissue was slightly tough in consistency, with a similar grayish-white to grayish-red appearance on sectioning.

**Figure 3:**
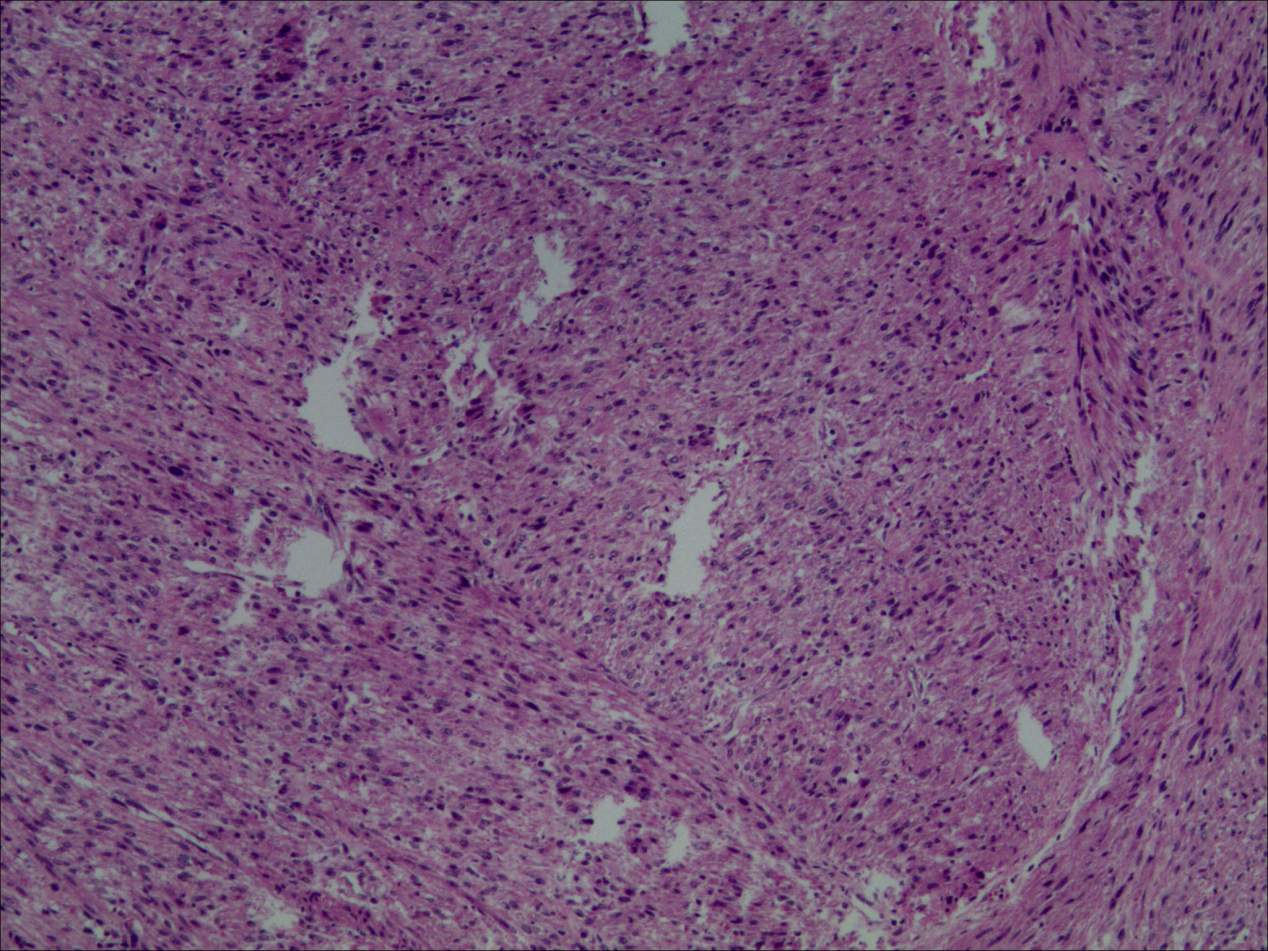
Light microscopic examination of the tumor post-first resection (H&E stain, 10x10) revealed spindle-shaped cells arranged in fascicles. These cells exhibited indistinct boundaries, mild nuclear atypia, and slightly coarse chromatin. Additionally, bizarre nuclei were scattered, and mitotic figures ranged from 0 to 1 per 10 high power fields. Infarction was observed in some tumor cells.

**Figure 4:**
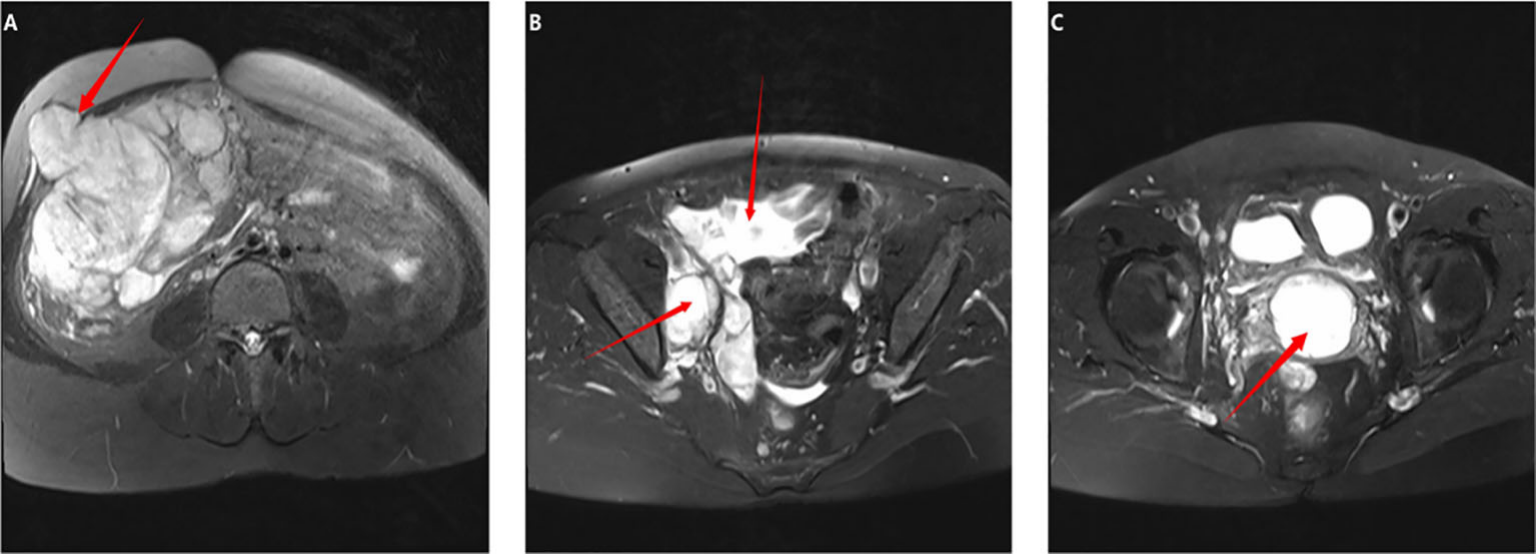
Imaging findings prior to the second surgery: **(A–C)** Whole abdomen MRI: **(A, B)** Multiple space-occupying lesions were identified in the right lower abdomen and pelvic cavity. The largest lesion, located in the lower abdomen, measured 11.6x10.6x8.4 cm and locally protruded into the right abdominal wall, involving the right rectus abdominis muscle. It exhibited isointense and hypointense signals on T1-weighted images and a slightly hyperintense signal on T2-weighted images. **(C)** A mass measuring approximately 3.6x4.2x4.2 cm was observed at the vaginal stump, displaying a hypointense signal on T1-weighted and a slightly hyperintense signal on T2-weighted images.

On December 14, 2021, we performed a total laparoscopic hysterectomy and bilateral salpingectomy on a patient. During surgery, the uterus resembled that of a five-month pregnancy in size and was irregularly shaped with multiple fibroids, the largest being 12 cm in diameter on the anterior wall. The uterus was removed transvaginally without protective measures. The general picture is as follows (**Figure 2**). Microscopic examination post-surgery revealed the mass to be a leiomyoma, with most areas infarcted and some showing bizarre nuclei and rare mitotic figures (0-1/10 HPF) (**Figure 3**). The patient was advised of the need for close follow-up. A month post-operation, transabdominal ultrasonography showed no abnormalities. However, four months later, she developed abdominal distension. Repeat ultrasonography identified several heterogeneous masses of varying sizes in the right pelvic and abdominal regions, the largest measuring 12.5x11.8x7.8 cm. Further MRI revealed multiple abnormal signal shadows involving the vaginal stump, right lower quadrant, omentum, mesentery, intestinal space, pelvic cavity, and areas near the common iliac vessel bifurcation and rectum. A lesion in the right lower quadrant had penetrated the right transversus abdominis muscle, reaching the subcutaneous fat (**Figure 4**). Serum CA12-5 and LDH levels were elevated to 46.4 U/mL and 340 U/L, respectively, while other markers remained normal. Physical examination revealed significant abdominal distention with a large, palpable mass extending three fingers above the umbilicus and showing limited mobility. A firm, cystic mass approximately 5 cm in diameter was palpable at the vaginal stump, although the vaginal mucosa was intact.

**Figure 5:**
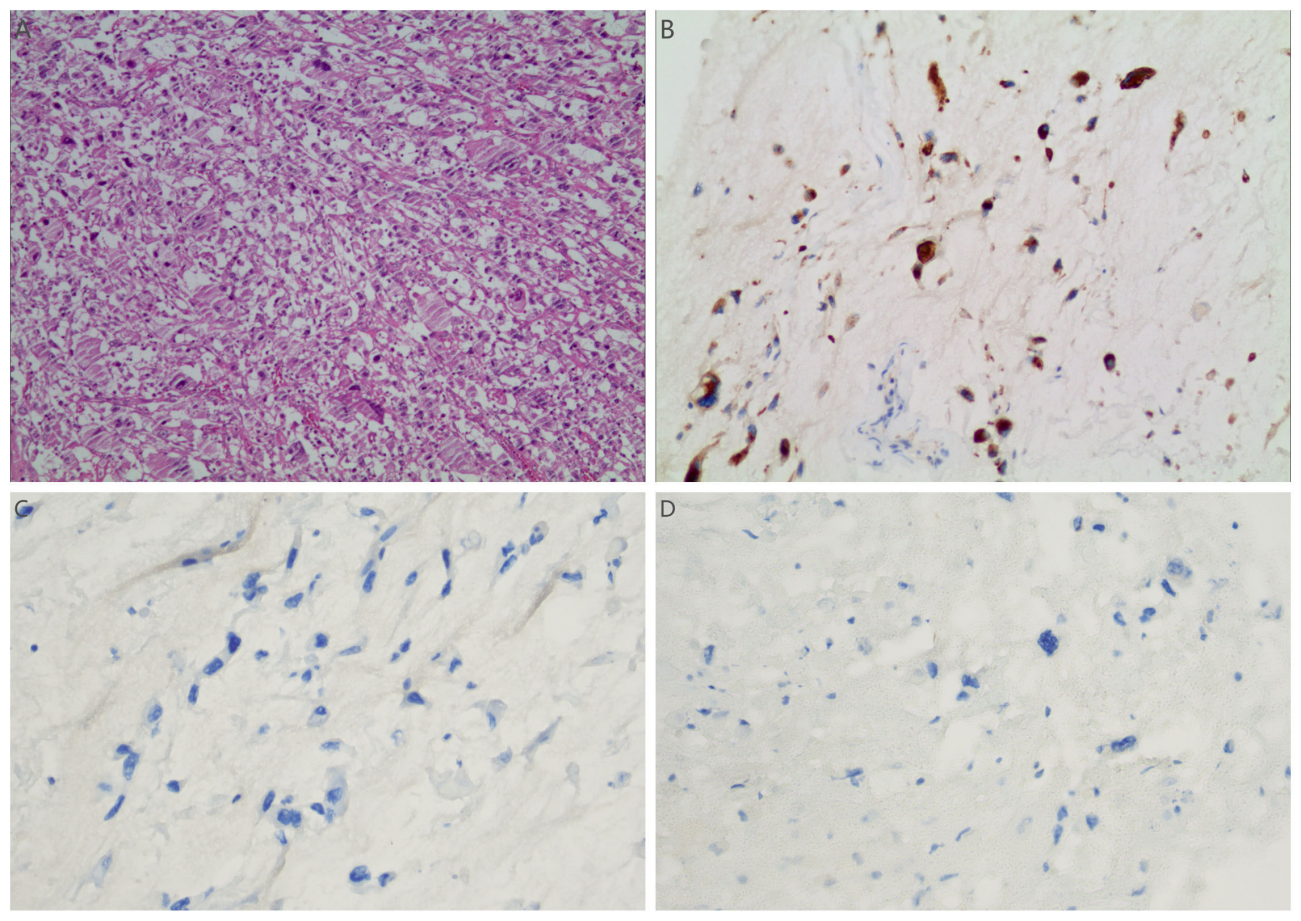
**(A)** Light microscopic examination of the tumor post-second resection (H&E stain, 10x10) showed pleomorphic cells with indistinct boundaries. The cytoplasm varied from eosinophilic to clear. There was severe nuclear atypia, prominent nucleoli, and conspicuous mitotic figures. The immunohistochemical results of sarcoma component: **(B–D)** tumor cells showed positive for Vimentin **(B)**, negative for SMA **(C)** and Desmin **(D)**.

On April 29, 2022, we performed an exploratory laparotomy, including resections of pelvic, abdominal, and mesenteric tumors, and a palliative resection of the right colon tumor. During surgery, we identified a 20 cm tumor in the abdominal cavity, covered by the greater omentum and prone to bleeding, with complex, primarily mucous internal tissue. The tumor encased parts of the ascending and transverse colon, as well as the ileum, and extended through the right peritoneum to the right rectus abdominis muscle. Additional tumors up to 3 cm in diameter were found on the small intestine and descending colon, and larger tumors up to 7 cm were present in the pelvis. Enlarged retroperitoneal lymph nodes were noted, the largest measuring 4 cm. The bilateral ovaries were not visible. We advised the patient’s family of the challenging intraoperative conditions and the impossibility of complete cytoreductive surgery, recommending a palliative approach instead. They consented to this plan. Microscopic analysis confirmed undifferentiated sarcomas in the abdominal and pelvic masses, with tumor involvement noted in the right colon, part of the ileum, mesentery and its lymph nodes, omentum, and appendix (**Figure 5**). Postoperatively, the patient experienced pleural effusion, right hydronephrosis, and poor incision healing. After symptomatic treatment, her condition improved. Considering her overall status, we recommended follow-up chemotherapy. However, the patient chose to discontinue treatment for personal reasons and was discharged, resulting in unsuccessful follow-up.

## 3 Discussion

Morcellation, a surgical technique, involves dividing tissue into smaller fragments to facilitate removal through tiny incisions or with laparoscopic tools, thereby reducing the size of the uterus or myomas. Historically, gynecologists employed manual morcellation via the vagina to extract an enlarged uterus (10). Power morcellation, introduced in 1993, shaves or cuts tissue to facilitate tissue extraction (11). Studies have shown that both power morcellation and manual segmentation increase the risk of recurrence (12, 13). Anupama et al. (14) and Della Badia et al. (15) research demonstrated that the inappropriate use of fibroids in patients with uterine sarcoma can promote tumor metastasis and even result in patient mortality. Small visible and microscopic tissue fragments may scatter throughout the abdomen following morcellation. As a consequence, tumor tissue may disseminate to the peritoneum as a result of this process (16). The meta-analysis comprised 24 studies involving 384 patients, investigating the prognosis of patients with uterine leiomyosarcoma across various morcellation methods. The findings indicated that patients undergoing electric morcellation exhibited the poorest prognosis, with a 5-year survival rate of 30%, whereas those without morcellation demonstrated the most favorable prognosis, boasting a 5-year survival rate of 60%. Patients subjected to cold knife morcellation (e.g., electrocautery or scissors) exhibited an intermediate prognosis, achieving a 5-year survival rate of 59% (17). The laparoscopic removal of uterine fibroids has garnered attention following the issuance of a safety communication warning by the FDA in 2014 (1). In 2017, the European Society of Gynecological Oncology Statement (ESGO) and the US FDA 2020 recommended employing closed shredding bags. These bags aim to facilitate minimally invasive techniques while preventing tissue dissemination in the abdominal cavity (17, 18). These measures are effective in preventing the dissemination of tumor tissue.

The previously described case involved the removal of a tumor that was initially fragmented into small pieces using a scalpel within the vagina. Following the initial operation, the tumor rapidly developed extensive pelvic and abdominal metastases, prompting an analysis of potential causes. Firstly, the malignancy risk associated with the uterine tumor was not thoroughly evaluated before the initial operation. Secondly, the dissemination of lesions resulted from the fragmentation of the uterus without protective measures. Thirdly, the initial pathological diagnosis of uterine sarcoma was imprecise, exacerbated by the impact of morcellation on pathological assessment. The lack of further treatment post-operation significantly affected the prognosis. Therefore, adherence to the tumor-free principle in vaginal tumor excision is crucial. While the vagina serves as an optimal natural passage for uterine extraction, difficulties arise in extracting large uteri from narrow vaginas, necessitating techniques such as segmentation, cutting, and splitting. Previous studies indicate that even intravaginal shredding with a protective bag fails to prevent tumor spread due to potential bag leakage during vaginal morcellation (19). Thus, our attention should be directed towards the shredding technique itself, regardless of the method of extraction.

Measures should be implemented to maximize the benefits of morcellation while minimizing the risk of tumor dissemination associated with it. Specific patient screening for suitability for morcellated surgery is imperative. However, there is currently no reliable preoperative diagnostic method for uterine sarcoma (17). This presents a considerable challenge for gynecologists. Patients undergoing laparoscopic hysterectomy (myomectomy) require thorough evaluation regarding the malignancy risk of uterine tumors prior to surgery. As specialists, we must focus on identifying clinical risk factors for uterine sarcoma. These factors include the following (5): Patients were predominantly perimenopausal and postmenopausal women, with uterine sarcoma typically diagnosed between the ages of 50 and 60, while cases under 40 were relatively uncommon. The patient presented with abnormal uterine bleeding, indicative of rapid fibroid growth, particularly notable in postmenopausal women not undergoing hormone replacement therapy. Additionally, the patient had a medical history of tamoxifen use and pelvic radiation therapy, as well as a childhood history of retinoblastoma and hereditary leiomyomatosis and renal cell carcinoma (HLRCC). Furthermore, further imaging examinations, cervical cancer screenings, and endometrial tissue sampling are necessary to identify malignancies (17). MRI can assess tumor invasion into the uterine muscles and surrounding structures, as well as lymph node metastasis, which are typical manifestations of uterine sarcoma on MRI (20–22). MRI imaging of uterine sarcoma typically reveals a large solid mass with an indistinct boundary and heterogeneous signal intensity. Uterine sarcomas exhibit abnormally high signals on T2-weighted imaging (T2WI), whereas uterine fibroids often display low signals. Additionally, uterine sarcomas may demonstrate unclear borders and infiltrative growth patterns. A slightly elevated signal on T1-weighted imaging (T1WI) suggests intra-mass hemorrhage and necrosis. During dynamic contrast-enhanced MRI (DCE-MRI), the mass exhibits early enhancement, while the necrotic areas remain unenhanced. Diffusion-weighted imaging (DWI) typically reveals restricted diffusion within the sarcoma due to dense tumor cellularity, resulting in decreased apparent diffusion coefficient (ADC) values. Nonetheless, there are overlapping MRI features between uterine sarcoma and atypical uterine fibroids (23), therefore, it is crucial to integrate detailed clinical information with T1WI, T2WI, DWI, and DCE-MRI for preoperative diagnosis to avoid missing patients with potential uterine sarcoma. While patients with uterine sarcoma do not exhibit specific tumor markers, elevated serum levels of LDH and CA12-5, as demonstrated in some studies, hold diagnostic significance for uterine sarcoma (24, 25). Despite thorough preoperative evaluation, it is essential to thoroughly discuss all available treatment options, along with their respective benefits and drawbacks, with patients considering laparoscopic hysterectomy. Provide patients with information on diverse surgical approaches for managing uterine fibroids, empowering them to make informed decisions.

Undoubtedly, intraoperative tumor-free technique remains pivotal for preventive measures. Emphasizing the closed morcellation bag’s role as a safety measure in laparoscopic hysterectomy (myoma) is crucial, representing an integral component of morcellation. However, during total laparoscopic hysterectomy, where an enlarged uterus is extracted from the vagina, there has been limited advocacy for protective sleeves. Under such circumstances, morcellation could theoretically lead to the dissemination of undetected malignant cells (17). Based on this case, it is imperative to adhere to the tumor-free principle, irrespective of whether tumor removal occurs from the abdomen, vagina, or elsewhere. Recognizing this concern, some studies suggest placing the uterus in a specimen bag before vaginal extraction if tissue fragmentation is required post laparoscopic hysterectomy (26). Recent studies have introduced a novel technique for vaginal morcellation within a closed environment. This approach offers both vaginal protection and retraction while securely containing the specimen to prevent loss during morcellation, thereby minimizing the risk of unforeseen malignancy (27).

Morcellation disrupts the structural integrity of tissue specimens, posing specific challenges for intraoperative pathological diagnosis and surgical pathological staging (28). Research indicates that morcellation frequently disrupts the junctional region between endometrial stromal or smooth muscle tumors and the surrounding myometrium. However, histological examination of this junctional region is crucial for the classification of atypical stromal tumors (29). Following uterine morcellation, accurately assessing the size of the uterine tumor and the depth of myometrial infiltration by epithelial or stromal lesions becomes challenging. This may have contributed to the incomplete or even missed pathological diagnosis during the initial operation of this case.

## 4 Conclusions

In conclusion, while morcellation can benefit patients, it can also lead to complications. To prevent similar tragedies, whether removing morcellated tumor tissue from the vagina or the abdomen, the concept of maintaining a tumor-free environment should be further emphasized and the application of tumor-free technology should be strengthened.

## Data Availability

The original contributions presented in the study are included in the article/supplementary material. Further inquiries can be directed to the corresponding author.
